# Oligomerization by co-assembly of β-amyloid and α-synuclein

**DOI:** 10.3389/fmolb.2023.1153839

**Published:** 2023-03-20

**Authors:** Jin Ryoun Kim

**Affiliations:** Department of Chemical and Biomolecular Engineering, New York University, 6 MetroTech Center, Brooklyn, NY, United States

**Keywords:** alpha-synuclein, aggregation, beta-amyloid, oligomer, protein-protein interaction

## Abstract

Aberrant self-assembly of an intrinsically disordered protein is a pathological hallmark of protein misfolding diseases, such as Alzheimer’s and Parkinson’s diseases (AD and PD, respectively). In AD, the 40–42 amino acid-long extracellular peptide, β-amyloid (Aβ), self-assembles into oligomers, which eventually aggregate into fibrils. A similar self-association of the 140 amino acid-long intracellular protein, α-synuclein (αS), is responsible for the onset of PD pathology. While Aβ and αS are primarily extracellular and intracellular polypeptides, respectively, there is evidence of their colocalization and pathological overlaps of AD and PD. This evidence has raised the likelihood of synergistic, toxic protein-protein interactions between Aβ and αS. This mini review summarizes the findings of studies on Aβ-αS interactions related to enhanced oligomerization *via* co-assembly, aiming to provide a better understanding of the complex biology behind AD and PD and common pathological mechanisms among the major neurodegenerative diseases.

## 1 Introduction

**FIGURE 1:**
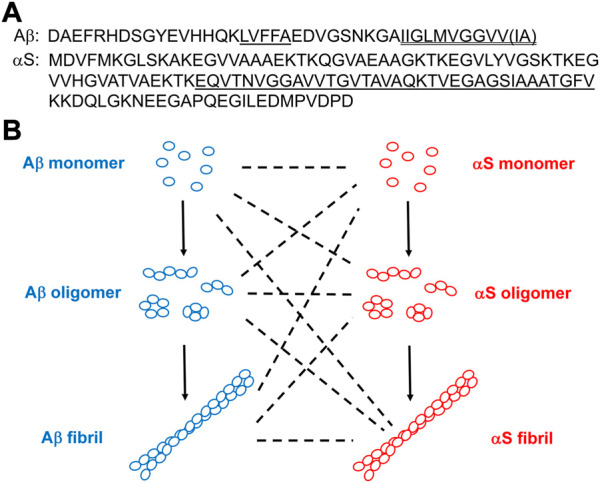
**(A)** Amino acid sequences of Aβ and αS. In Aβ, the hydrophobic Aβ17-21 (LVFFA) and the hydrophobic Aβ C-terminus are shown single-underlined and double-underlined, respectively. The two additional C-terminal residues of Aβ42 relative to Aβ40 are Ile-Ala shown in a parenthesis. In αS, the NAC domain is underlined. **(B)** Schematics of aggregations of Aβ and αS by self-assembly (solid lines) from their monomeric forms to oligomeric, and to fibrillar forms or *via* co-assembly (dotted lines).

Alzheimer’s disease (AD) is the most common neurodegenerative disorder characterized by the losses of forebrain cholinergic and hippocampal neurons (Dallaire-Theroux et al., 2017). AD pathology is caused by aggregation of the peptide, β-amyloid (Aβ), containing 40 or 42 residues (referred to as Aβ40 and Aβ42, respectively; Figure 1A) leading to the formation of extracellular amyloid plaques in the brains of AD patients (Aguzzi and O'Connor, 2010). The typical physiological ratio of Aβ40/Aβ42 is ∼9 (Qiu et al., 2015). The hydrophobic sequences of Aβ (e.g., Aβ17-21 and the C-terminus; Figure 1A) promote Aβ aggregation (Bolognesi et al., 2010; Hu et al., 2012). Aβ aggregation involves three distinct conformers (Figure 1B): monomeric Aβ spontaneously self-assembles into soluble oligomeric Aβ (Gao et al., 2020; Xiao et al., 2020), which then aggregates further to form insoluble fibrillar Aβ (Petkova et al., 2002; Luhrs et al., 2005). The primary toxic agents in AD are oligomeric rather than monomeric or fibrillar Aβ (Kayed et al., 2003; Aguzzi and O'Connor, 2010). Toxic Aβ oligomers can disrupt neuronal activity at the synapse, disturb cell membranes, cause oxidative stress, and perturb calcium homeostasis (Aguzzi and O'Connor, 2010), thereby initiating degeneration in AD (He et al., 2019). While Aβ monomers and some small Aβ oligomers are structurally disordered (Jin et al., 2011; Potapov et al., 2015), β-sheet content grows with increasing size of oligomeric Aβ (Ono et al., 2009). The major Aβ oligomeric morphologies include spherical and protofibrillar forms (Harper et al., 1997; Lasagna-Reeves et al., 2011). Aβ fibrils adopts in-register cross β-sheet structures (Petkova et al., 2002; Luhrs et al., 2005).

The AD afflicted brain is also characterized by intraneuronal filamentous aggregates composed of phosphorylated tau (Twohig and Nielsen, 2019), a microtubule-binding protein that consists of 352–441 residues. The multiple repeat regions in tau are aggregation-prone (Jouanne et al., 2017). In AD, tau pathology generally occurs later relative to neuronal cell loss, memory deficit and Aβ plaque formation (Oddo et al., 2003) and can be induced by Aβ aggregates (Gotz et al., 2001; Vasconcelos et al., 2016).

Parkinson’s disease (PD) is the second most common neurodegenerative disorder after AD, and the most common movement disorder (Surmeier et al., 2017). PD is characterized by the loss of substantia nigra dopamine neurons and the presence of Lewy bodies (LB) with intracellular protein inclusions that contain the 140 residue-protein, α-synuclein (αS; Figure 1A) (Irwin et al., 2013). Hydrophobic αS 61–95, known as the non-amyloid component (NAC; Figure 1A), is critical in αS self-assembly (Giasson et al., 2001). Similar to the role of Aβ in AD, aggregation of αS is the hallmark event in PD pathology (Surmeier et al., 2017; Hijaz and Volpicelli-Daley, 2020). Self-assembly of αS monomers produces soluble αS oligomers, which eventually aggregate into fibrillar αS (Figure 1B). As is the case with Aβ in AD, oligomeric rather than monomeric or fibrillar αS is a major toxic agent in PD (Winner et al., 2011; Chen et al., 2015). Toxic αS oligomers disrupt lipid membranes (Giehm et al., 2011; Winner et al., 2011), disturb ion homeostasis (Danzer et al., 2007) and cause oxidative stress (Cremades et al., 2012). Many toxic αS oligomers are β sheet-structured and globular or pore-like in morphology (Giehm et al., 2011; Cremades et al., 2012). Also, like Aβ, monomeric αS is structurally disordered (Eliezer et al., 2001) and fibrillar αS is cross β-sheet-structured (Vilar et al., 2008).

The recent progresses in AD therapy and diagnosis have reinforced the critical role of oligomers in AD pathology, though no symptom-modifying therapeutic agents are currently available for PD (Paolini Paoletti et al., 2020). A couple of antibody-based AD drugs, aducanumab and lecanemab, have recently been approved by FDA (Walsh et al., 2021; Couzin-Frankel and Piller, 2022). Aducanumab targets Aβ aggregates, including oligomers and fibrils (Sevigny et al., 2016), whereas lecanemab does Aβ protofibrillar oligomers (van Dyck et al., 2023). These findings support that amyloid oligomers play a critical role in neurodegenerative diseases, though the efficacy of aducanumab and the safety of lecanemab are still debatable (Walsh et al., 2021; Couzin-Frankel and Piller, 2022). While oligomerizations of single amyloid proteins have been extensively examined (for example, see (Nguyen et al., 2021)), oligomer formation driven by interactions between multiple amyloid proteins (e.g., Aβ and αS) is relatively understudied, which is a major focus of this minireview.

## 2 Pathological synergy between AD and PD *via* interactions between Aβ and αS

A great deal of evidence indicates that both AD- and PD-related symptoms are detected in some patients. For example, many (∼50%) patients with AD develop LB pathology in addition to Aβ plaques and tau tangles (Irwin et al., 2013; Visanji et al., 2019). These patients exhibit faster cognitive decline and shorter lifespan compared to those with only AD pathology (Kraybill et al., 2005). Similarly, PD patients can be diagnosed with dementia (PDD) and PDD patients show more severe cognitive dysfunction than AD patients (Hamilton 2000; Irwin et al., 2013). Moreover, both dementia and parkinsonism are evident in Diffuse Lewy Body disease (DLB) (Hamilton 2000). Importunately, previous *in vivo* studies with transgenic (Tg) mice showed greater neuronal degeneration, stronger neuroinflammation, and more severe deficits in cognition and motor skill when Aβ and αS are both present (Larson et al., 2017; Khan et al., 2018; Lloyd et al., 2021). These findings serve as direct evidence that the synergistic connections of AD and PD pathologies are mediated by Aβ and αS (Twohig and Nielsen, 2019; Bassil et al., 2020; Murakami and Ono, 2022).

## 3 Oligomerization enhanced by Aβ-αS interactions: Clinical and animal studies

Strong evidence in the literature has shown that the synergistic toxic effects are associated with enhanced oligomerization *via* Aβ-αS interactions. For instance, the level of soluble αS oligomers is higher and amyloid plaque load is lower in human brain with AD and PD co-pathologies than in AD alone (Heyman et al., 1999; Tsigelny et al., 2008). A post-mortem analysis showed greater amounts of toxic oligomeric αS in AD brains compared to healthy controls (Larson et al., 2017). Aβ and αS co-expression in double Tg mice enhances αS oligomerization compared to single Tg mice, while reducing Aβ fibrillar plaques (Larson et al., 2017; Khan et al., 2018). Moreover, intracerebral injections of αS-containing brain extracts into AD mice inhibit Aβ deposition into fibrillar plaques, presumably increasing the quantity of Aβ oligomers (Bachhuber et al., 2015).

## 4 Co-localization of Aβ and αS for direct protein-protein interactions

While Aβ and αS can facilitate oligomerization of each other indirectly, for example, by enhancing protein production (Roberts et al., 2017), a large body of evidence points to direct Aβ-αS interactions as a more effective cross-talk mechanism (Ren et al., 2019). Generally, Aβ and αS are considered as extracellular and intracellular proteins, respectively. Nevertheless, a chance of these proteins being in spatial proximity is not negligible, permitting the direct protein-protein interactions. For example, expression levels of αS are high in human brain regions where AD lesions are abundant (Mandal et al., 2006). In addition, co-localization of Aβ and αS (either its NAC fragment or full-length form) was detected within fibrillar plaques (Drummond et al., 2017; Bluhm et al., 2022) in clinical and animal studies and suggested to occur in cellular compartments, such as an mitochondrion (Hashimoto et al., 2003). Aβ once produced extracellularly can be accumulated intraneuronally (LaFerla et al., 2007). Likewise, αS initially produced intracellularly can be secreted into extracellular space (Lee et al., 2005). Thus, direct protein-protein interactions between Aβ and αS occur both intracellularly and extracellularly (Masliah et al., 2001; Hashimoto et al., 2003; Tsigelny et al., 2008; Kayed et al., 2020).

## 5 Impact of Aβ-αS interaction on aggregation: *In vivo* and *in vitro* studies

Studies on Aβ-αS interactions *in vivo* would be most ideal to understand the outcome of the interactions under a biological context. Several animal and cell culture studies have reported impact of direct Aβ-αS interactions on aggregations (Table 1). For example, Aβ promotes αS oligomerization and LB inclusions in Tg mice and cultured neurons (Masliah et al., 2001; Tsigelny et al., 2008; Larson et al., 2017; Khan et al., 2018; Lloyd et al., 2021). Aβ plaque deposition can be accelerated by αS in animal studies (Clinton et al., 2010), though the opposite effects of αS were reported elsewhere (Bachhuber et al., 2015; Khan et al., 2018; Lloyd et al., 2021). Unfortunately, conformations of amyloid proteins are crucial for their associations with other amyloid proteins, internalization mechanisms and pathological effects (Peng et al., 2018; Kayed et al., 2020), yet are difficult to control in samples of biological origin (Peng et al., 2018). Similarly, the collection of brain-derived amyloid aggregates requires sample treatments that can alter aggregation state (Casali and Lanreth, 2016), for example, the use of denaturing agents (e.g., Sarkosyl) for extraction (Ren and Sahara, 2013) or acidic buffer for elution of proteins (Puangmalai et al., 2020). Moreover, isolations of Aβ and αS from biological origins suffer from low quantities and the lack of concentration controls (Casali and Lanreth, 2016).

**TABLE 1:** Summary on effects of Aβ-αS interactions on aggregation.

Effect on aggregation	Model system	Experimental method	References
Aβ enhances αS fibrillar neuronal inclusions	APP/αS mice and αS mice	Immunohistochemistry; Transmission electron microscopy	Masliah et al. (2001)
	APP/αS mice and αS mice with injection of recombinant αS (F)	Immunohistochemistry	Lloyd et al. (2021)
	Synthetic Aβ42 (fresh) and neuronal cells expressing αS	Immunostaining	Masliah et al. (2001)
Aβ promotes αS oligomerization	APP/αS mice and αS mice	Immuno-dot blot assay; Western blot	Masliah et al. (2001), Tsigelny et al. (2008), Larson et al. (2017), Khan et al. (2018)
	Synthetic Aβ42 (fresh and aggregated) and recombinant αS (fresh)	Western blot	Masliah et al. (2001), Tsigelny et al. (2008)
Aβ promotes αS aggregation	Synthetic Aβ42 (fresh, O and F) and recombinant αS (fresh)	Thioflavin T fluorescence	Ono et al. (2012), Koppen et al. (2020)
αS promotes Aβ plaque deposition	APP/αS_A53T_ mice and APP mice	ELISA; Thioflavin S fluorescence; Immunohistochemistry	Clinton et al. (2010)
αS inhibits Aβ plaque deposition	APP/αS mice and APP mice	Immunofluorescence	Khan et al. (2018)
	APP/αS mice and APP mice with injection of recombinant αS (F)	Immunohistochemistry	Lloyd et al. (2021)
	APP/αS_A30P_ mice and APP mice	Immunofluorescence	Bachhuber et al. (2015)
	APP mice with injection of αS_A30P_-containing brain extracts	Immunofluorescence	Bachhuber et al. (2015)
αS promotes Aβ aggregation	Synthetic Aβ40 or Aβ42 (fresh) and recombinant αS (O and F)	Thioflavin T fluorescence; Transmission electron microscopy	Ono et al. (2012)
αS inhibits Aβ aggregation	Synthetic Aβ42 (fresh) and recombinant αS (fresh)	Thioflavin T fluorescence	Bachhuber et al. (2015)
αS inhibits Aβ fibrillization	Synthetic Aβ40 or Aβ42 (M and O) and recombinant αS (M, O and F)	Native-PAGE with in-gel fluorescence; Competitive immuno-dot blot assay	Candreva et al. (2020), Chau and Kim (2022)
αS promotes Aβ oligomerization	Synthetic Aβ40 or Aβ42 (M and O) and recombinant αS (M, O and F)	Native-PAGE with in-gel fluorescence; Competitive immuno-dot blot assay	Candreva et al. (2020), Chau and Kim (2022)

APP/αS mice: double Tg mice expressing human APP and human αS

APP mice: single Tg mice expressing human APP

αS mice: single Tg mice expressing human αS

αS_A30P_: αS variant with A30P mutation

αS_A53T_: αS variant with A53T mutation

M: monomer

O: oligomer

F: fibril

Fresh: freshly prepared

Instead, *in vitro* studies with synthetic Aβ and recombinant αS might be more suitable to examine Aβ-αS interactions for several reasons: 1) synthetic Aβ and recombinant αS injections into animal models have proven effective for studying the onset, progression and outcomes of AD and PD pathologies, and their co-pathology (Bloom 2014; McAllister et al., 2020); 2) conformations and aggregation states of Aβ and αS − critical for the severity and the phenotype of pathology − are relatively easier to control and characterize *in vitro* than *in vivo* (McAllister et al., 2020), permitting an understanding of molecular outcomes from specific Aβ-αS interactions. In previous *in vitro* studies (Table 1), Aβ enhanced αS aggregation (Ono et al., 2012; Koppen et al., 2020), including αS oligomerization (Masliah et al., 2001; Tsigelny et al., 2008). αS can either accelerate (Ono et al., 2012) or inhibit Aβ aggregation *in vitro* (Bachhuber et al., 2015). Unfortunately, the intrinsic complexity associated with different conformers of Aβ and αS (i.e., monomeric, oligomeric, and fibrillar forms) has precluded the identification of exact synergistic mechanisms linked to AD, PD and their co-pathologies. Moreover, most previous studies have provided limited insight into conformer-specific Aβ-αS interactions. This limitation arose because aggregation was frequently ill-defined with no distinction between oligomerization and fibrilization. In addition, Aβ-αS interactions were often characterized under denaturing conditions (e.g., with SDS), which can introduce undesired artifacts on aggregation states, complicating data interpretation. Other histopathology and injection studies have focused on fibrils, underestimating the role of oligomers in comorbidities.

## 6 Conformer-specific Aβ-αS interactions: αS-assisted oligomerization of Aβ *in vitro*

**FIGURE 2:**
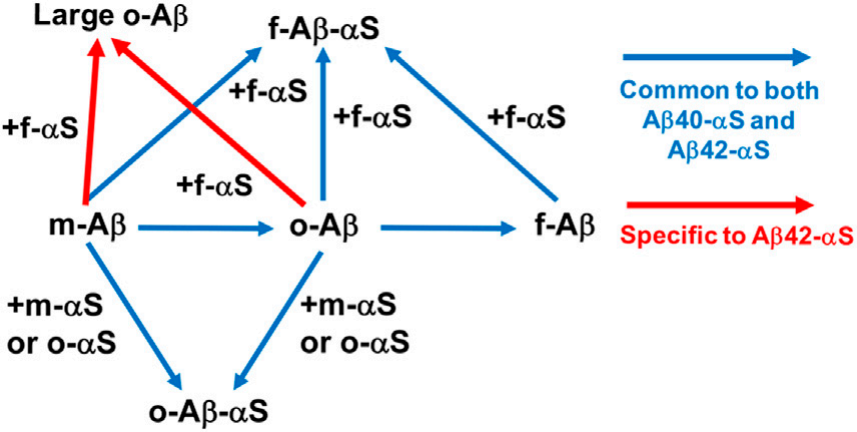
Direct Aβ-αS interaction networks. Blue arrows: common to both Aβ40-αS and Aβ42-αS (Candreva et al., 2020; Chau and Kim, 2022). Addition of αS monomers and oligomers inhibits fibrilization of monomeric (m-) and oligomeric (o-) Aβ, while promoting oligomerization of Aβ monomers and stabilizing preformed Aβ oligomers *via* co-assembly. Monomeric, oligomeric, and fibrillar (f-) Aβ can be incorporated into αS fibrils. Red arrows: specific to Aβ42-αS (Chau and Kim, 2022). αS fibrils partially interferes with conversion of soluble Aβ42, but not Aβ40, into insoluble aggregates, instead possibly promoting the formation of large oligomeric Aβ42.

Recent *in vitro* studies by Kim and coworkers have provided experimental evidence of Aβ-αS co-assembly into potentially toxic oligomers, revealing important insights into the nature of Aβ-αS interactions: excess soluble αS species (i.e., αS monomers and oligomers) prevent fibrillization of Aβ40 while enhancing oligomerization of Aβ40 (Figure 2; Table 1). Though the αS monomer concentration tested in this study was 350 μM, exceeding the physiological αS concentrations (i.e., low μM range (Wilhelm et al., 2014)), oligomeric αS at 17 μM, which is slightly above the physiological αS concentrations, was effective for enhancing Aβ oligomerization *in vitro*. Thus, this observation suggests a possibility of oligomeric αS seeding Aβ oligomerization *in vivo*. Moreover, *in vivo* αS concentrations can be elevated locally by various mechanisms (Owen et al., 2019), which may promote the formation of αS oligomers. Once formed, these oligomers are kinetically stable at < μM concentrations (Nag et al., 2011), available for enhancing Aβ oligomerization. In the same study, the C-terminus of Aβ40 was shown to be directly involved in interactions with αS, facilitating the formation of co-assembled oligomers. The competitive immunoassay provides evidence for binding of Aβ40 in all three forms (i.e. monomeric, oligomeric and fibrillar Aβ40) to αS fibrils *via* the Aβ C-terminus (Candreva et al., 2020). In addition, aggregations of αS monomers and oligomers were facilitated by Aβ40 fibrils (Candreva et al., 2020), as reported elsewhere (Ono et al., 2012). Interestingly, when judged by thioflavin T fluorescence, incorporation of αS into fibrillar Aβ40 or Aβ40 into fibrillar αS was relatively minor or slow compared to co-assembled oligomerization (Candreva et al., 2020). The implication is that Aβ40-αS interactions might occur more drastically for the formation of oligomers rather than fibrils.

The subsequent study showed both similarities and dissimilarities between Aβ42-αS and Aβ40-αS interactions. Specifically, while monomeric and oligomeric αS promoted oligomerization of Aβ42, similar to Aβ40, αS fibrils induced the formation of large Aβ42 oligomers - a finding unique to Aβ42 (Figure 2; Table 1) (Chau and Kim, 2022). The C-terminus of Aβ42 is primarily utilized for its interactions with αS, similar to Aβ40, yet other regions of Aβ42 (e.g., Aβ22-35) are also involved (Chau and Kim, 2022). A molecular dynamics simulation study suggested that direct Aβ42-NAC interactions induce the formation of new β-strands in Aβ42 while the NAC domain remains structurally unchanged (Atsmon-Raz and Miller, 2016). A similar structural study with Aβ and the full-length αS will provide valuable insight into the molecular determinants crucial in Aβ-αS interactions.

Overall, these studies demonstrate that Aβ and αS can directly interact to form Aβ-αS co-assembled oligomers, possibly responsible for the enhanced oligomerization *in vivo* when Aβ and αS co-exist. Thus, Aβ-αS interactions provide a synergistic mechanism to produce oligomeric assemblies and may have common biological consequences in AD, PD, DLB and PDD, given that amyloid oligomers are usually neurotoxic (Ahmed et al., 2010; Giehm et al., 2011).

## 7 Discussion

### 7.1 Aβ-αS interactions in a biological context

For a better understanding of pathogenic overlap between AD and PD, how the conformer-specific Aβ-αS interactions are manifested in a biological context would be a critical first step (Murakami and Ono, 2022). This biological examination would interrogate the pathological relevance of Aβ-αS interactions, closing a knowledge gap in a link among Aβ-αS co-assembly, oligomerization and synergistic toxic effects. Intracellular and extracellular co-localizations of Aβ and αS (Chinta et al., 2010; Drummond et al., 2017) raise a possibility of transportation across cell membranes of oligomers formed by Aβ-αS interactions, spreading their toxic effects (Kayed et al., 2020). Given αS’s ubiquitous and abundant expression in brain and strong innate ability to shuttle between intra- and extracellular compartments (Irwin et al., 2013), the role of αS in orchestrating a pathophysiological ensemble of AD and PD by enhancing Aβ oligomerization is of a particular therapeutic relevance (Kayed et al., 2020). Moreover, the co-assembly of Aβ and αS may facilitate circumvention of their proteolytic clearances, possibly increasing their toxic effects *in vivo*. Thus, a strategy to modulate the interactions would open a new therapeutic avenue, beyond targeting self-assembly of a single amyloid protein adopted by most anti-amyloid therapeutic strategies developed against AD and PD (Murakami and Ono, 2022).

### 7.2 Additional complexity

While Aβ-αS interactions may provide additional routes to form oligomeric species beyond aggregation of individual amyloid proteins, heterogeneity of oligomers in size, morphology, structure, and seeding ability further increases pathological complexity. Aβ and αS oligomers of biological or synthetic origins range from dimers to ∼200 mers (Breydo and Uversky, 2015) and display different morphologies, such as globular and protofibrillar shapes (Chen et al., 2015). While many Aβ and αS oligomers possess well-defined secondary structures, usually β-sheets, others are structurally disordered (Breydo and Uversky, 2015; Gao et al., 2020). Moreover, amyloid oligomers can be either on-pathway intermediates of fibrillization or off-pathway end products (Breydo and Uversky, 2015), differing in seeding ability. Inhibitions of Aβ42 fibrillization by off-pathway αS oligomers - generated by forming covalent adducts with a dopamine metabolite or a polyunsaturated fatty acid - have recently been reported (Dhakal et al., 2021), though whether the modulations lead to Aβ-αS co-assembly into oligomers remains unclear. In this study, interactions of Aβ42 with the two structurally distinct off-pathway αS oligomers were found to promote the formation of Aβ42 assemblies that differ in toxicity, suggesting the importance of αS oligomer conformation in the observed phenotypes and clinical manifestations (Dhakal et al., 2021). Thus, testing with specific subgroups of Aβ and αS oligomers for the outcomes of their interactions would further depict the heterogeneity-driven complexity of the interactions.

The Aβ-αS interaction may extend further with other amyloidogenic and non-amyloidogenic proteins, increasing the boundaries of interaction networks (Murakami and Ono, 2022). For example, tau is an intracellular amyloid protein, whose filamentous aggregates are found in the AD afflicted brain (Buee et al., 2000; Twohig and Nielsen, 2019). Tau is highly abundant as soluble monomers and does not spontaneously aggregate under the physiological condition (Jouanne et al., 2017; Visanji et al., 2019), yet its aggregation into oligomers and filaments related to neurotoxicity can be induced by Aβ following phosphorylation-dependent and independent mechanisms (Jin et al., 2011; Vasconcelos et al., 2016; Gyparaki et al., 2021). Similar to Aβ and αS, oligomeric rather than monomeric or filamentous tau is toxic (Ghag et al., 2018) and responsible for neuronal dysfunction in AD (Spires et al., 2006). αS can also induce tau aggregation in both phosphorylation-dependent (Twohig and Nielsen, 2019) and independent manners (Giasson et al., 2003). αS oligomers rather than αS monomers and fibrils catalyze tau oligomerization (Lasagna-Reeves et al., 2010) and αS and tau can promote their mutual aggregations (Giasson et al., 2003; Dasari et al., 2019). Thus, the direct Aβ-αS interactions extend further with tau through the shared release, trafficking and uptake mechanisms (Twohig and Nielsen, 2019; Visanji et al., 2019), that allow intra- and inter-cellular propagation of pathological seeds of Aβ, αS and tau (Frost et al., 2009; Irwin et al., 2013; Kayed et al., 2020), initiating an autocatalytic cycle of aggregation (Vasconcelos et al., 2016) and spreading pathology (Lloyd et al., 2021). The protein-protein interaction network centered on Aβ-αS can also extend with a non-amyloidogenic protein, such as DNA-binding protein TDP-43, known to bind to αS (Dhakal et al., 2022).

## 8 Conclusion

Aβ-αS interactions leading to the formation of co-assembled oligomers and their propagation are deemed responsible for pathological complexity, overlap, and heterogeneity of AD, PD and other major neurodegenerative diseases. Thus, a better understanding of Aβ-αS interactions will be required to close current knowledge gaps and point to new therapeutic strategies targeting oligomerization.
